# Root-tip-mediated inhibition of hydrotropism is accompanied with the suppression of asymmetric expression of auxin-inducible genes in response to moisture gradients in cucumber roots

**DOI:** 10.1371/journal.pone.0189827

**Published:** 2018-01-11

**Authors:** Nobuharu Fujii, Sachiko Miyabayashi, Tomoki Sugita, Akie Kobayashi, Chiaki Yamazaki, Yutaka Miyazawa, Motoshi Kamada, Haruo Kasahara, Ikuko Osada, Toru Shimazu, Yasuo Fusejima, Akira Higashibata, Takashi Yamazaki, Noriaki Ishioka, Hideyuki Takahashi

**Affiliations:** 1 Graduate School of Life Sciences, Tohoku University, Katahira, Aoba-ku, Sendai, Japan; 2 Japan Space Forum, Kanda-Surugadai, Chiyoda-ku, Tokyo, Japan; 3 Faculty of Science, Yamagata University, Kojirakawa-machi, Yamagata, Japan; 4 Advanced Engineering Services Co., Ltd., Takezono, Tsukuba, Japan; 5 JEM Utilization Center, Japan Aerospace Exploration Agency, Sengen, Tsukuba, Japan; 6 Japan Manned Space Systems Co., Ltd., Otemachi, Chiyoda-ku, Tokyo, Japan; 7 Graduate School of Medicine, Teikyo University, Kaga, Itabashi-ku, Tokyo, Japan; 8 Institute of Space and Astronautical Science, Japan Aerospace Exploration Agency, Yoshinodai, Sagamihara, Japan; University of Massachusetts Amherst, UNITED STATES

## Abstract

In cucumber seedlings, gravitropism interferes with hydrotropism, which results in the nearly complete inhibition of hydrotropism under stationary conditions. However, hydrotropic responses are induced when the gravitropic response in the root is nullified by clinorotation. Columella cells in the root cap sense gravity, which induces the gravitropic response. In this study, we found that removing the root tip induced hydrotropism in cucumber roots under stationary conditions. The application of auxin transport inhibitors to cucumber seedlings under stationary conditions suppressed the hydrotropic response induced by the removal of the root tip. To investigate the expression of genes related to hydrotropism in de-tipped cucumber roots, we conducted transcriptome analysis of gene expression by RNA-Seq using seedlings exhibiting hydrotropic and gravitropic responses. Of the 21 and 45 genes asymmetrically expressed during hydrotropic and gravitropic responses, respectively, five genes were identical. Gene ontology (GO) analysis indicated that the category auxin-inducible genes was significantly enriched among genes that were more highly expressed in the concave side of the root than the convex side during hydrotropic or gravitropic responses. Reverse transcription followed by quantitative polymerase chain reaction (RT-qPCR) analysis revealed that root hydrotropism induced under stationary conditions (by removing the root tip) was accompanied by the asymmetric expression of several auxin-inducible genes. However, intact roots did not exhibit the asymmetric expression patterns of auxin-inducible genes under stationary conditions, even in the presence of a moisture gradient. These results suggest that the root tip inhibits hydrotropism by suppressing the induction of asymmetric auxin distribution. Auxin transport and distribution not mediated by the root tip might play a role in hydrotropism in cucumber roots.

## Introduction

Plants are sessile organisms that have evolved tropisms to regulate the growth direction of their organs in response to environmental cues, such as gravity and moisture gradients, causing the organs to grow towards more suitable environmental conditions. Roots bend downward via gravitropism and bend towards wetter areas of the soil via hydrotropism (reviewed in [1]). Under stationary conditions, the roots of wild-type pea exhibit very weak hydrotropism, whereas the roots of an agravitropic pea mutant exhibit strong hydrotropism [2]. When wild-type pea seedlings were rotated on a 3D clinostat and the roots were exposed to a moisture gradient, the roots bent to the wetter side of the gradient [3]. When cucumber seeds were planted in water-absorbent plastic in which the roots would emerge into the air space and germinated under microgravity conditions, the elongated lateral roots slanted towards the side of the water-absorbent plastic foam, which was located on the opposite side of the growth direction of the primary root [4]. The lateral roots were slanted downward towards the ground, and when the seedlings were rotated using a 3D clinostat, the lateral roots slanted towards the side containing water-absorbent plastic foam, indicating that the lateral roots of cucumber seedlings can exhibit hydrotropism and that the hydrotropism of the lateral roots is usually suppressed by gravity responses [4]. In addition, the 3D clinostat experiment suggested that the primary roots of cucumber seedlings can induce hydrotropism when they are placed perpendicularly, but not in parallel, to the direction of the moisture gradient [5]. These findings suggest that gravity responses suppress root hydrotropism in pea and cucumber seedlings. In contrast to pea and cucumber, the roots of *Arabidopsis thaliana*, rice, and *Lotus japonicus* can exhibit hydrotropism under stationary conditions by overcoming gravitropism [6, 7]. Thus, the strength of the hydrotropic response and its interaction with gravitropism differ among plant species.

The Cholodny-Went hypothesis states that the plant hormone auxin is differentially redistributed in response to gravistimulation and causes asymmetrical growth [8]. When Arabidopsis roots are placed in a horizontal position, the auxin efflux carriers AtPIN3/AtPIN7 relocalize onto the lower sides of columella cells and excrete auxin to the lower side of the root cap [9, 10]. Auxin excreted from columella cells is thought to be transported through epidermal cells to the elongation region via AtPIN2 [11]. Thus, more auxin accumulates in the lower side than in the upper side of the root, leading to downward bending of the root.

The possible role of auxin in root hydrotropism has also been investigated. In cucumber roots, the auxin-inducible gene *CsIAA1* is asymmetrically expressed during hydrotropism as well as gravitropism [5]. These findings suggest that the asymmetric redistribution of auxin causes differential growth during hydrotropism, which is known to occur during gravitropism. Inhibitors of auxin efflux carriers were recently shown to inhibit root hydrotropism as well as root gravitropism in cucumber seedlings [12]. Furthermore, CsPIN5 protein, an ortholog of AtPIN2, differentially accumulates in roots during the hydrotropic response, with higher accumulation in the wet side than in the dry side [12]. In contrast to cucumber roots, hydrotropically bending Arabidopsis roots do not asymmetrically express the auxin marker gene, *DR5*:*uidA* [1]. In addition, agravitropic mutants of Arabidopsis such as *aux1* and *pin2*, which are defective in auxin influx and efflux carriers, respectively, exhibit normal hydrotropism [6]. Moreover, inhibitors of auxin influx and efflux carriers inhibit gravitropism, but not hydrotropism, in Arabidopsis roots [13]. These findings suggest that the dependency of root hydrotropism on auxin transport differs among plant species.

Removing root columella cells by laser ablation in Arabidopsis roots and surgically removing the root cap in maize roots reduce gravitropism, suggesting that columella cells sense gravity [14–16]. By contrast, removing root columella cells in Arabidopsis roots by laser ablation does not inhibit hydrotropism [17]. In rice roots, removing a 0.2-mm-long section of the root tip did not inhibit hydrotropism, but it inhibited gravitropism [7]. These results suggest that Arabidopsis and rice roots without root tip cells can sense moisture gradients and exhibit hydrotropism. These findings prompted us to examine the effects of root tip removal on hydrotropism in cucumber roots, as hydrotropism is suppressed by gravitropism in cucumber roots and the root tip plays a crucial role in root gravitropism. In the current study, we found that removing the root tip induced root hydrotropism in cucumber seedlings under stationary conditions. We then examined the effects of auxin transport inhibitors on the induction of root hydrotropism by removing root tips under stationary conditions. We also performed transcriptome analysis via RNA-Seq to identify genes asymmetrically expressed during hydrotropic as well as gravitropic responses in intact cucumber roots, and examined the expression of these genes in de-tipped roots exhibiting hydrotropism. Our results provide new insights into how the gravity response interferes with hydrotropism in cucumber roots.

## Materials and methods

### Plant growth and root curvature measurements

Seeds of cucumber (*Cucumis sativus* L.) cv. Shinkasyuu-jibai were purchased from Watanabe Seed Co. (Kogota, Miyagi, Japan). Root hydrotropism was induced in cucumber seedlings as previously described [5], with minor modifications. Seven cucumber seeds were inserted into a crack created in water-absorbent plastic foam and enclosed in a plastic container for 18 h under stationary conditions. After germination, a saturated solution of K_2_CO_3_ was injected into the filter paper placed at the opposite side of the roots in the container to induce a moisture gradient in the plastic container (S1 Fig). For the control, cucumber roots were placed under water-saturated conditions, with all inner sides of the plastic container covered with Extra Thick (2.45 mm) Blot Absorbent Filter Papers (Bio-Rad, Hercules, CA, USA) saturated with distilled water. The seedlings were grown in the dark at 26 ± 1°C. Rotation on a two-axis (3D) clinostat can remove gravity responses from the plants and simulate plant growth under microgravity conditions [18–20]. To rotate the cucumber seedlings on a 3D clinostat, the plastic containers were fixed on a metal plate that rotates on two axes using packaging tape and rotated at 2 rpm. The cucumber roots were cut in half longitudinally to separate the wet (concave) and dry (convex) sides after exposing them to a moisture gradient for 4 h. As a control, immediately before exposure to hydrostimulation, the seeds were germinated and the resulting seedlings were grown for 18 h under stationary conditions. Roots of these seedlings were cut longitudinally into halves exposed to the water-absorbent plastic side or the opposite side of the container. For convenience, the side of the system with water-absorbent plastic foam and the opposite side are referred to as the wet side and dry side, respectively. As another control, 18-h-old cucumber seedlings were grown under H_2_O-saturated conditions for 4 h, after which the roots were longitudinally divided into the half grown on the wet side versus the dry side. Under these conditions, the roots randomly bent. Therefore, roots that bent to the wet side and those that bent to the dry side were separately harvested, and each root was independently used for analysis.

To gravistimulate the cucumber roots, eight seeds were vertically placed into a crack created in a block of water-absorbent plastic foam (45 × 20 × 10 mm). The two blocks with cucumber seeds were attached to the inner surface of a plastic cap and sealed in a plastic container (60 × 60 × 60 mm) after being supplied with distilled water so that the seedling roots could be aeroponically grown in humid air. After incubating the seedlings in a vertical position (at a right angle to the horizon) for 24 h at 26 ± 1°C, the seedlings were further grown in a vertical position for the control. To gravistimulate the seedlings, the plastic container was re-oriented to a horizontal position (parallel to the horizon) and further incubated.

After exposing the cucumber roots to a moisture gradient or to gravistimulation, the seedlings were photographed with a digital camera (EOS20D, Canon, Tokyo, Japan, or E-10, Olympus, Tokyo, Japan). The seedlings were chemically fixed by pulling them with the water-absorbent plastic foam from the plastic container and incubating them in fixative solution (4% [w/v] paraformaldehyde and 0.25% [v/v] glutaraldehyde in 50 mM sodium phosphate buffer [pH 7.2]) at 4°C overnight. Each root was imaged under a stereoscopic microscope (SZX16, Olympus) using a digital camera (DP70, Olympus). The images were used to measure root curvature with ImageJ software (http://imagej.nih.gov/ij/). For RNA isolation, the water-absorbent plastic foam with cucumber seedlings was incubated in RNAlater (Thermo Fisher, Waltham, MA, USA) at 4°C overnight.

To remove the root tip, 18-h-old seedlings were detached from the water-absorbent plastic foam and placed under a stereoscopic microscope (SZX16, Olympus). The root tip (size 0.5 mm) containing columella cells and a quiescent center was removed using a micro blade (Microfeather Blade, Feather Safety Razor Co., Ltd., Osaka, Japan). Seedlings without root tips were placed into the water-absorbent plastic foam and returned to the chamber for further incubation under the designated conditions.

The stock solutions of 2,3,5-triiodobenzonic acid (TIBA) (Sigma-Aldrich, St. Louis, MO, USA) and 9-hydroxyfluorene-9-carboxylic acid (HFCA) (Sigma-Aldrich) were prepared at 10^−1^ M in dimethyl sulfoxide (DMSO; Wako, Tokyo, Japan). Cucumber seeds were pretreated with the inhibitor solutions (10^−4^ M) in 0.1% (v/v) DMSO by immersion for 3 h. The pretreated seeds were then placed in the plastic foam blocks containing 10 mL of the same inhibitor solution, and hydrotropism assays were conducted as described earlier.

### Gene expression analysis

Root tip sections (0.5 mm) were removed under a stereomicroscope (SZX16, Olympus) and discarded. The remaining 1.5 mm root proper was cut in half longitudinally using a micro blade (Microfeather Blade). Each half of an identical root was separately placed into RNAlater (Thermo Fisher) and incubated at 4°C overnight. Seven cucumber seedlings were used to prepare one RNA sample. Harvested samples were immediately analyzed or stored at -80°C until use. Total RNA was isolated using an RNeasy Plant Mini Kit (Qiagen, Inc., Valencia, CA, USA). The total RNA was treated with DNase I (RNase-Free DNase Set, Qiagen, Inc.), followed by an RNeasy MinElute Cleanup Kit (Qiagen, Inc.). RNA integrity (RIN) was checked with an RNA 6000 Nano Assay Kit and Agilent 2100 Bioanalyzer, and RNA that showed RIN value >6.2 was used for RNA-Seq. RNA-Seq to analyze gene expression during root hydrotropism in cucumber seedlings was performed by Takara Bio Inc. (Kusatsu, Shiga, Japan). To analyze gene expression during root gravitropism in cucumber seedlings, RNA-Seq was supported and conducted by KAKENHI (Grant-in-Aid for Scientific Research) in the Priority Area "Comparative Genomics" from the Ministry of Education, Culture, Sports, Science and Technology of Japan. Libraries for sequencing were constructed using standard methods (TruSeq RNA Sample Prep Kit provided by Illumina, San Diego, CA, USA). All RNA-Seq was conducted on an Illumina HiSeq™ 2500. The resulting RNA-Seq data in this article can be found in the DNA Data Bank of Japan (DDBJ) Sequence Read Archive (DRA) under DRA Accession number DRA006032 for cucumber root hydrotropism data and DRA Accession number DRA003914 for cucumber root gravitropism data. After obtaining the sequencing data in FASTQ format provided by Illumina HiSeq, adaptor sequences and low-quality sequences were removed using cutadapt (version 1.1; http://code.google.com/p/cutadapt/) and Trimmomatic (version 0.32; http://www.usadellab.org/cms/?page=trimmomatic), respectively. The resulting sequences were mapped to the cucumber genome sequences (cucumber_ChineseLong_v2_genome.fa; http://www.icugi.org/cgi-bin/ICuGI/genome/home.cgi?ver=2&organism=cucumber&cultivar=Chinese-long) with TopHat (version 2.0.9; http://tophat.cbcb.umd.edu/), together with Bowtie (version 1.0.0; http://bowtie-bio.sourceforge.net/index.shtml) and SAMtools (version 0.1.19; http://samtools.sourceforge.net/). The mapping results were used to calculate FPKM (Fragments Per Kilobase of transcript per Million mapped reads) values, representing mRNA levels, with Cuffdiff, which is included in the Cufflinks package (version 2.1.1; http://cole-trapnell-lab.github.io/cufflinks/). Gene ID is the gene identification number for the cucumber_ChineseLong_v2_genome in the Cucurbit Genomics Database [21, 22]. Annotation is according to the cucumber_ChineseLong_v2_genome of the Cucurbit Genomics Database [21, 22] or genome-wide identification of auxin response-related gene families in cucumber [23], or is the result of searching with Basic Local Alignment Search Tool on NIH (https://blast.ncbi.nlm.nih.gov/Blast.cgi). Gene Ontology (GO) analysis was conducted using the website (http://www.icugi.org/cgi-bin/ICuGI/tool/GO_enrich.cgi) on the Cucurbit Genomics Database using the dataset from cucumber Chinese long v2 and *P*-value correction methods for False Discovery Rate (FDR). GO terms for which the corrected *P*-value was <0.01 were considered to be significantly enriched among asymmetrically expressed genes.

To examine gene expression by reverse transcription followed by quantitative polymerase chain reaction (RT-qPCR), cDNA was synthesized by incubating a mixture of 1 μL of RNA (100 ng) that was heat-denatured at 65°C (1 min), 2 μL of ReverTra Ace qPCR RT Master Mix (Toyobo, Osaka, Japan), and 7 μL of H_2_O at 37°C (15 min), 50°C (5 min), and 98°C (5 min). To conduct qPCR, 10 μL of SYBR Green Super mix (Bio-Rad), 1 μL of forward and reverse primers (5 μM each), 6 μL of H_2_O, and 2 μL of cDNA synthesized from 4 ng of RNA were combined. The mixture was exposed to 45 cycles of two steps comprising 95°C (5 sec) for denaturation and 55°C (1 min) for primer annealing/primer extension after denaturation at 95°C (3 min). The samples were then subjected to melt-curve analysis using the MyiQ Single Color Real-Time PCR Detection System (Bio-Rad). The gene-specific primers used for PCR are shown in S1 Table. Biologically independent experiments were repeated three or more times for analysis. Each biologically independent experiment was used to calculate relative gene expression levels between the sides of roots or among treatments of root segments to obtain a mean value of 1.0 using the relative ratio of threshold cycle (Ct) values between the specific gene and the F-actin-capping protein subunit alpha gene (Csa2M031720), which was constitutively expressed in our experiments. The resulting relative gene expression levels were used to calculate the means and SD and to analyze their statistical significance.

To examine the effects of auxin on gene expression, cucumber seedlings were grown on wet filter paper at 26 ± 1°C in the dark and subjected to gene expression analysis when their roots were 2 mm long. After removing the root tip (0.5 mm), a 1.5 mm segment was excised from the root and treated with auxin according to the methods of [5, 24]. To deplete endogenous auxin, the excised roots were incubated for 1.5 h in 15 mM sucrose containing 50 μg mL^-1^ chloramphenicol, followed by 30 min in incubation buffer (1 mM citrate, 1 mM PIPES, 15 mM sucrose, 1 mM KCl, 50 μg mL^-1^ chloramphenicol, pH 6.0). After auxin starvation, the root segments were stored in incubation buffer with or without IAA for 2 h. The root segments were stored in RNAlater (Thermo Fisher) at 4°C overnight prior to analysis.

### Statistical analysis

To compare the relative gene expression levels between one side of the root and the other during root tropism, Student’s *t* test was performed using Microsoft Excel software (Microsoft Japan Co. Ltd., Tokyo, Japan). When relative gene expression levels were compared among root segments in each treatment group and when root curvature or root length was compared, the data were first analyzed by one-way ANOVA using “anova”, a default command in R, version 3.1.3 (http://www.r-project.org; R Development Core Team, Boston, MA, USA), and then the data in which differences were detected were compared by the Tukey method using “Tukey HSD”, a default command in R.

## Results

### Effects of de-tipping on hydrotropism in cucumber roots

**Fig 1:**
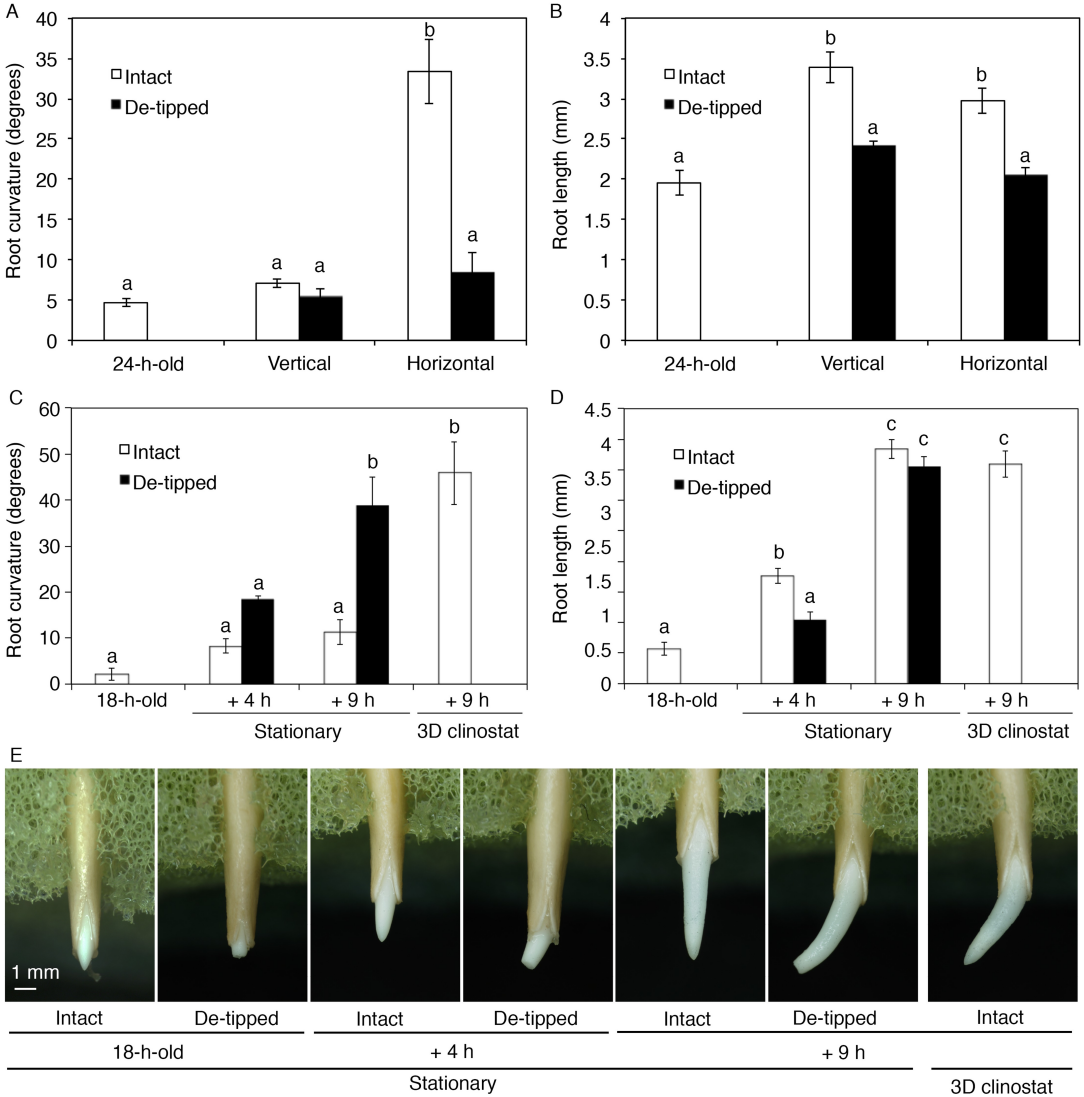
Effects of root tip removal on root hydrotropism in cucumber seedlings. (A–B) Effects of root tip removal on root gravitropism in cucumber seedlings. 24-h-old cucumber seedlings in which the root tip was either not removed (Intact) or removed (De-tipped) were placed in a vertical or horizontal position. After 1 h, root curvature (A) and length (B) were measured. Experiments (using eight seedlings) were repeated three times, and the mean ± SE was calculated. (C–E) Effects of root tip removal on root hydrotropism in cucumber seedlings. 18-h-old cucumber seedlings in which the root tip was either not removed (Intact) or removed (De-tipped) were exposed to a moisture gradient induced by K_2_CO_3_. After incubation under stationary conditions for 4 h and 9 h or in a 3D clinostat for 9 h, root curvature (C) and length (D) were measured. Experiments (using seven seedlings) were repeated three times, and the mean ± SE was calculated. Different letters indicate statistically significant differences (*P*<0.01), which were analyzed by one-way ANOVA and Tukey HSD tests.

Columella cells in the root cap sense gravity, thereby regulating auxin transport to determine the direction of root growth [9, 10, 14–16]. Ablating root columella cells with a laser reduces gravitropism, but not hydrotropism, in Arabidopsis roots [17], and the surgical removal of a 0.2-mm-long segment of the root tip reduces gravitropism but not hydrotropism in rice roots [7]. These findings piqued our interest in the effects of surgically removing the cucumber root tip on root hydrotropism, because gravitropism interferes with hydrotropism in cucumber roots and because the root tip (which contains columella cells) plays a crucial role in root gravitropism. First, the effects of de-tipping of cucumber roots on root gravitropism were confirmed. Placing the roots, from which the root tip (0.5 mm) had been removed, in a horizontal position for 1 h resulted in a reduction in gravitropic curvature (Fig 1A). This result is consistent with previous findings for other plant species such as maize and rice [7, 14, 15]. At 1 h after the root tip was removed, de-tipped roots were shorter than intact roots (Fig 1B). It should be noted that gravitropism of the de-tipped roots was partially recovered within 4 h following seedling re-orientation (S2 Fig).

Intact roots of cucumber seedlings did not display distinct hydrotropism under stationary conditions, although a hydrotropic response was observed when the seedlings were clinorotated in the presence of a moisture gradient (Fig 1C and 1E). When the root tip (0.5 mm in length) was removed, roots exposed to a moisture gradient under stationary conditions for 9 h significantly bent to the wet side (Fig 1C and 1E). Hydrotropic curvature of the de-tipped roots did not significantly differ from that of intact roots that had been clinorotated in the presence of a moisture gradient (Fig 1C and 1E). At 4 h and 9 h after the root tip was removed, root length of de-tipped roots became to be similar to that of intact roots (Fig 1D). These results indicate that reducing root gravitropism by removing the root tip de-represses root hydrotropism in cucumber seedlings grown under stationary conditions.

**Fig 2:**
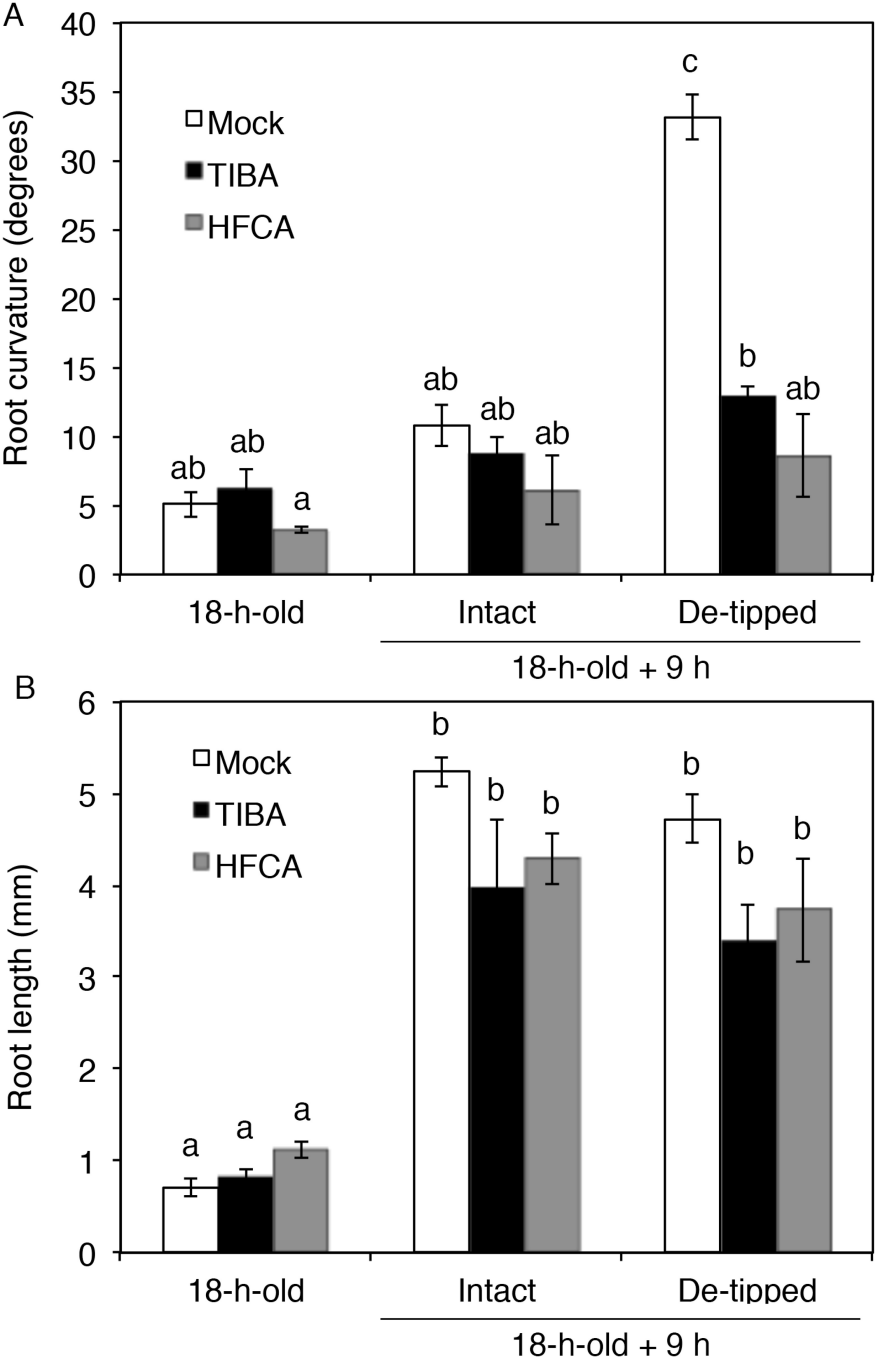
Effects of auxin transport inhibitors on cucumber root hydrotropism under stationary conditions induced by removing the root tip. The cucumber seeds were germinated in a vertical position without (Mock) or with auxin transport inhibitors (TIBA or HFCA) for 18 h. 18-h-old cucumber seedlings in which the root tip was either not removed (Intact) or removed (De-tipped) were exposed to a moisture gradient induced by K_2_CO_3_. After incubation under stationary conditions for 9 h without (Mock) or with auxin transport inhibitors (TIBA or HFCA), root curvature (A) and length (B) were measured. Experiments using seven cucumber seedlings were repeated three times, and the results represent the mean ± SE. Different letters indicate statistically significant differences (*P*<0.01), which were analyzed by one-way ANOVA and Tukey HSD tests.

Recently, auxin transport was proposed to be required for the hydrotropic response in cucumber roots, since auxin transport inhibitors reduce hydrotropism in cucumber [12]. We therefore examined whether auxin transport is required for de-tipping-inducible hydrotropism using the auxin transport inhibitors TIBA and HFCA (Fig 2). When de-tipped roots were treated with TIBA or HFCA, hydrotropism was significantly inhibited (Fig 2A). These results suggest that root hydrotropism induced by the removal of the root tip requires auxin transport.

### Transcriptome analysis to identify asymmetrically expressed genes

To examine whether root hydrotropism due to root tip removal under stationary conditions occurs in a manner similar to hydrotropism in intact cucumber roots, we attempted to identify asymmetrically expressed genes in intact cucumber roots during hydrotropism. We constructed 12 libraries (S2 Table) from cucumber roots exposed to various moisture conditions and obtained 51 to 72 million sequence reads per library. More than 95% of the reads from each library could be mapped to the cucumber reference genome sequence (cucumber_ChineseLong_v2_genome.fa) (S2 Table). After mapping the reads, we calculated FPKM values, representing the amount of mRNA per gene, and compared these values between one side of the root and the other. Table 1 lists the genes with asymmetric expression at a *Q*-value <0.05; *Q*-value is the *P*-value adjusted by FDR. When the roots were exposed to a moisture gradient in the K_2_CO_3_ chamber for 4 h under clinorotated conditions, 21 genes were asymmetrically expressed. Among these, 17 genes were more highly expressed in the wet side of the root than the dry side, whereas four genes were more highly expressed in the dry side than the wet side (Table 1). GO analysis indicated that 17 genes that were preferentially expressed in the wet side during root hydrotropism are specifically enriched in three GO terms: “auxin mediated signaling pathway”, “cellular response to auxin stimulus”, and “response to auxin stimulus” (S3 Table). These 17 genes include *CsIAA1*, which was previously found to be asymmetrically expressed during hydrotropic and gravitropic responses in cucumber roots [5]. Since Wu *et al*. in [23] renamed *CsIAA1* as *CsIAA12*, we refer to this gene as *CsIAA1(12)* hereafter.

**Table 1:** Asymmetrically expressed genes in cucumber roots exposed to moisture gradients, as determined by RNA-Seq.

Growth conditions^a^			Dir^b^	Gene ID	Annotation	FPKM^c^			*Q*-value	Pattern^d^	GO term analysis
						Wet side	Dry side	Wet/Dry			
0	-	-	-	None							
4	1G	K_2_CO_3_	-	Csa1G231530^e^	Aux/IAA protein, CsIAA24	35.93	15.75	2.28	0.045	Wet > Dry	No hit
				Csa7G419610	S-adenosylmethionine synthase	28.11	0.00	-	0.045		
				Csa6G445770	Phenylalanine ammonia-lyase	0.35	2.38	0.15	0.045	Wet < Dry	Hit
				Csa7G041870	Auxin induced-like protein	1.71	8.47	0.20	0.045		
				Csa6G445760	Phenylalanine ammonia-lyase	1.05	5.21	0.20	0.045		
				Csa1G534750	Chitinase 7	8.67	28.82	0.30	0.045		
				Csa3G446120	ABC transporter	1.87	5.45	0.34	0.045		
				Csa7G312940	Extensin	2.64	7.39	0.36	0.045		
				Csa7G432470	Blue copper protein	5.03	13.21	0.38	0.045		
				Csa5G215120	40S ribosomal protein	158.83	352.09	0.45	0.045		
				Csa2G006880^f^	Blue copper protein	12.71	26.49	0.48	0.045		
				Csa3G150000	Xyloglucan-specific endoglucanase inhibitor protein	16.98	33.52	0.51	0.045		
				Csa1G042560^f^	Lipid transfer protein	151.15	293.75	0.51	0.045		
				Csa5G374730	Auxin transporter-like protein 3 (CsLAX3)	64.90	119.58	0.54	0.045		
				Csa6G514890^f^	Pectinesterase	27.59	50.20	0.55	0.045		
				Csa4G285800	Peroxidase	57.36	104.26	0.55	0.045		
				Csa5G609750	Protein MKS1	0.00	2.28	-	0.045		
				Csa1G627480	Uncharacterized protein	0.00	1.60	-	0.045		
		H_2_O	-	None							
	3D	K_2_CO_3_	Wet	Csa5G610430	Aux/IAA protein	14.38	1.28	11.20	0.038	Wet > Dry	Hit
				Csa1G039830	Cytochrome P450	30.51	4.08	7.47	0.038		
				Csa1G231530^e^	Aux/IAA protein, CsIAA24	83.91	16.97	4.94	0.038		
				Csa4G303070^e^	Unknown protein	203.78	48.93	4.16	0.038		
				Csa6G120930^e^	Calmodulin-like protein	267.16	73.91	3.61	0.038		
				Csa1G537400^e^	Serine/threonine-protein kinase, CsPID	41.84	12.30	3.40	0.038		
				Csa1G397130	AUX/IAA protein, CsIAA13	22.83	7.79	2.93	0.038		
				Csa3G002330	WUSCHEL-related homeobox	10.83	4.16	2.60	0.038		
				Csa3G516530^e^	AUX/IAA protein, CsIAA1(12)	116.82	48.64	2.40	0.038		
				Csa3G176350	NAD(P)H-quinone oxidoreductase	192.75	80.63	2.39	0.038		
				Csa7G378520	Aux/IAA protein, CsIAA5	49.31	21.75	2.27	0.038		
				Csa7G048020	Pollen allergen-like protein	62.16	28.09	2.21	0.038		
				Csa5G215120	40S ribosomal protein	361.70	186.14	1.94	0.038		
				Csa5G161290	Nitrate transporter	83.19	44.45	1.87	0.038		
				CsaUNG024770	S-adenosylmethionine synthase	2.80	0.00	-	0.038		
				Csa7G070820	Unknown protein	3.16	0.00	-	0.038		
				Csa7G318970	Glycolipid transfer protein-related	1.25	0.00	-	0.038		
				Csa2G006880^f^	Blue copper protein	6.65	18.09	0.37	0.038	Wet < Dry	No hit
				Csa6G085120	Agglutinin-like protein	20.77	49.83	0.42	0.038		
				Csa6G514890^f^	Pectinesterase	17.94	35.12	0.51	0.038		
				Csa1G042560^f^	Lipid transfer protein	104.38	199.17	0.52	0.038		
		H_2_O	Wet	None							
			Dry	None							

^a^Growth conditions of time (h), gravitational conditions and moisture conditions for hydrostimulation are indicated.

^b^Direction of root bending is shown.

^c^FPKM (Fragments Per Kilobase of transcript per Million mapped reads) values representing mRNA levels.

^d^Asymmetric expression pattern is indicated.

^e^Gene ID is a gene identified by RNA-Seq as being asymmetrically expressed in gravitropically bending cucumber roots, as well as hydrotropically bending roots.

^f^Gene ID is a gene identified by RNA-Seq as being asymmetrically expressed in cucumber roots exposed to a moisture gradient under stationary conditions as well as in a 3D clinostat.

We also conducted RNA-Seq using the roots of 18-h-old seedlings prior to hydrostimulation, as well as roots that were further incubated under H_2_O-saturated conditions for 4 h. We failed to detect asymmetric gene expression supported by *Q*-value (less than 0.05) in either set of roots (Table 1). When the roots were exposed to a moisture gradient in the K_2_CO_3_ chamber under stationary conditions, the hydrotropic response was not observed because gravitropism counteracted the effects of hydrotropism. Under this condition, 18 genes were asymmetrically expressed between the wet and dry sides of the root (Table 1). Among these, 2 and 16 genes were preferentially expressed in the wet side and dry side, respectively. The 16 genes that were preferentially expressed in the dry side were specifically enriched in the GO terms “cell periphery” and “plasma membrane” in the Cellular Component Ontology and GO terms “phenylalanine ammonia-lyase activity” and “ammonia-lyase activity” in the Molecular Function Ontology (S4 Table). The genes in the latter two categories are two phenylalanine ammonia-lyase genes that are tandemly localized on cucumber chromosome 6.

In addition to root hydrotropism, we analyzed gene expression during root gravitropism. First, we analyzed the gravitropic curvature of cucumber roots in a time-course study (S3 Fig). Then, based on the curvature kinetics, we isolated RNA from the roots at 1 h after re-orienting the seedlings to the horizontal position, during which time the roots began to bend gravitropically. We extracted RNA from control roots at time zero and after further incubation for 1 h in a vertical position. We constructed six libraries (S5 Table) and obtained 23 to 33 million sequence reads per library for root gravitropism. More than 95% of the reads in each library could be mapped to the cucumber reference genome sequence (cucumber_ChineseLong_v2_genome.fa) (S5 Table).

We identified 45 asymmetrically expressed genes during root gravitropism by RNA-Seq (Table 2). Among these, 27 and 18 genes were preferentially expressed in the upper and lower sides of roots that had been re-oriented to the horizontal position, respectively. None of the 27 genes that were preferentially expressed in the upper side were assigned to specific GO terms by GO analysis (Table 2). Among the 18 genes preferentially expressed in the lower side, seven are *Aux/IAA* genes that were originally identified as auxin-inducible genes [24]. In addition to these *Aux/IAA* genes, a gene belonging to the *GH3* gene family that was also identified as an auxin-inducible gene [25] was preferentially expressed in the lower side. These 18 genes were specifically enriched in 32 GO terms and most of categorized genes were *Aux/IAA* genes (S6 Table). Among the genes preferentially expressed in the upper side, we identified an auxin-inducible *SAUR* gene [26].

**Table 2:** Asymmetrically expressed genes in gravitropic bending cucumber roots, as determined by RNA-Seq.

Growth conditions^a^		Gene ID	Annotation	FPKM^b^			*Q*-value	Pattern^c^	GO term analysis
				Lower side	Upper side	Lower/Upper			
0	-	None							
1	Vertical	None							
	Horizontal	Csa1G231530^d^	Aux/IAA protein, CsIAA24	53.27	2.88	18.52	0.02	Lower > Upper	Hit
		Csa3G516540	Aux/IAA protein, CsIAA25	49.43	7.12	6.94	0.02		
		Csa4G303070^d^	Unknown protein	95.79	14.15	6.77	0.02		
		Csa6G492310	Indole-3-acetic acid-amido synthetase, CsGH3.5	12.19	1.84	6.64	0.02		
		Csa3G002440	Unknown protein	44.64	9.47	4.71	0.02		
		Csa3G516530^d^	Aux/IAA protein, CsIAA1(12)	72.47	15.62	4.64	0.02		
		Csa6G507480	Protein of unknown function (DUF241, plant)	9.96	2.28	4.37	0.02		
		Csa1G537400^d^	Serine/threonine-protein kinase, CsPID	36.35	8.53	4.26	0.02		
		Csa1G627460	Polygalacturonase	13.81	3.63	3.81	0.02		
		Csa6G120930^d^	Calmodulin-like protein	258.50	72.30	3.58	0.02		
		Csa2G286540	Protein of unknown function (DUF4228)	73.38	23.99	3.06	0.02		
		Csa2G200440	AUX/IAA protein, CsIAA1	77.95	28.91	2.70	0.02		
		Csa3G177390	Protein of unknown function (DUF3475)	20.06	7.87	2.55	0.02		
		Csa7G378530	AUX/IAA protein, CsIAA3	122.66	48.14	2.55	0.02		
		Csa7G023920	Protein of unknown function (DUF241, plant)	44.07	17.95	2.46	0.02		
		Csa3G143580	Aux/IAA protein, CsIAA4	170.30	77.20	2.21	0.02		
		Csa7G440550	AUX/IAA protein	12.29	3.10	3.96	0.04		
		Csa6G516990	Unknown protein	626.93	166.16	3.77	0.04		
		Csa7G407500	Protein of unknown function (DUF761, plant)	0.00	2.01	-	0.02	Lower < Upper	No hit
		Csa6G109730	UDP-glycosyltransferase	0.00	0.47	-	0.02		
		Csa6G499150	Unknown protein	4.21	23.84	0.18	0.02		
		Csa3G826690	MYB family transcription factor-like protein	3.58	13.44	0.27	0.02		
		Csa5G149870	Retinol dehydrogenase 12	5.04	16.29	0.31	0.02		
		Csa3G629740	Polygalacturonase	7.12	22.63	0.31	0.02		
		Csa5G215140	LOB (Lateral organ boundaries) domain-containing protein, CsLBD22	28.43	88.60	0.32	0.02		
		Csa3G857600	Protein kinase C-like	6.99	21.59	0.32	0.02		
		Csa3G634290	Polygalacturonase	16.98	49.83	0.34	0.02		
		Csa2G416070	bZIP transcription factor family protein	33.16	97.17	0.34	0.02		
		Csa2G000750	Putative stress-induced protein (Protein of unknown function DUF1005)	12.29	35.47	0.35	0.02		
		Csa6G495830	Uridylyltransferase	2.59	7.43	0.35	0.02		
		Csa3G120410	Unknown protein	13.22	37.50	0.35	0.02		
		Csa2G258660	Auxin-induced SAUR-like protein, CsSAUR9	23.57	64.53	0.37	0.02		
		Csa2G367210	Cytokinin riboside 5'-monophosphate phosphoribohydrolase	12.44	32.79	0.38	0.02		
		Csa7G039260	Fasciclin-like arabinogalactan protein	109.60	274.79	0.40	0.02		
		Csa4G000030	Protein of unknown function (DUF1005)	10.88	26.92	0.40	0.02		
		Csa5G161870	Bifunctional inhibitor/plant lipid transfer protein/seed storage helical domain	75.28	184.48	0.41	0.02		
		Csa6G010000	N-hydroxycinnamoyl/ benzoyltransferase 4	24.08	57.87	0.42	0.02		
		Csa6G117780	Unknown protein	24.94	59.28	0.42	0.02		
		Csa2G011610	Protein of unknown function (DUF868, plant)	10.04	23.63	0.42	0.02		
		Csa3G088980	Pectinesterase	22.56	45.90	0.49	0.02		
		Csa3G743410	Putative zinc finger protein	2.96	10.43	0.28	0.04		
		Csa4G641590	Ethylene-responsive transcription factor	9.06	29.02	0.31	0.04		
		Csa2G003060	Unknown protein	18.88	43.15	0.44	0.04		
		Csa1G533440	Protein of unknown function (DUF4228)	40.20	90.36	0.44	0.04		
		Csa5G157230	Transcription factor bHLH	55.53	115.48	0.48	0.04		

^a^Growth conditions of time (h) and gravitational conditions for gravitstimulation are indicated.

^b^FPKM (Fragments Per Kilobase of transcript per Million mapped reads) values representing mRNA levels.

^c^Asymmetric expression pattern is indicated.

^d^Gene ID is a gene identified by RNA-Seq as being asymmetrically expressed in hydrotropically bending cucumber roots, as well as gravitropically bending roots.

**Fig 3:**
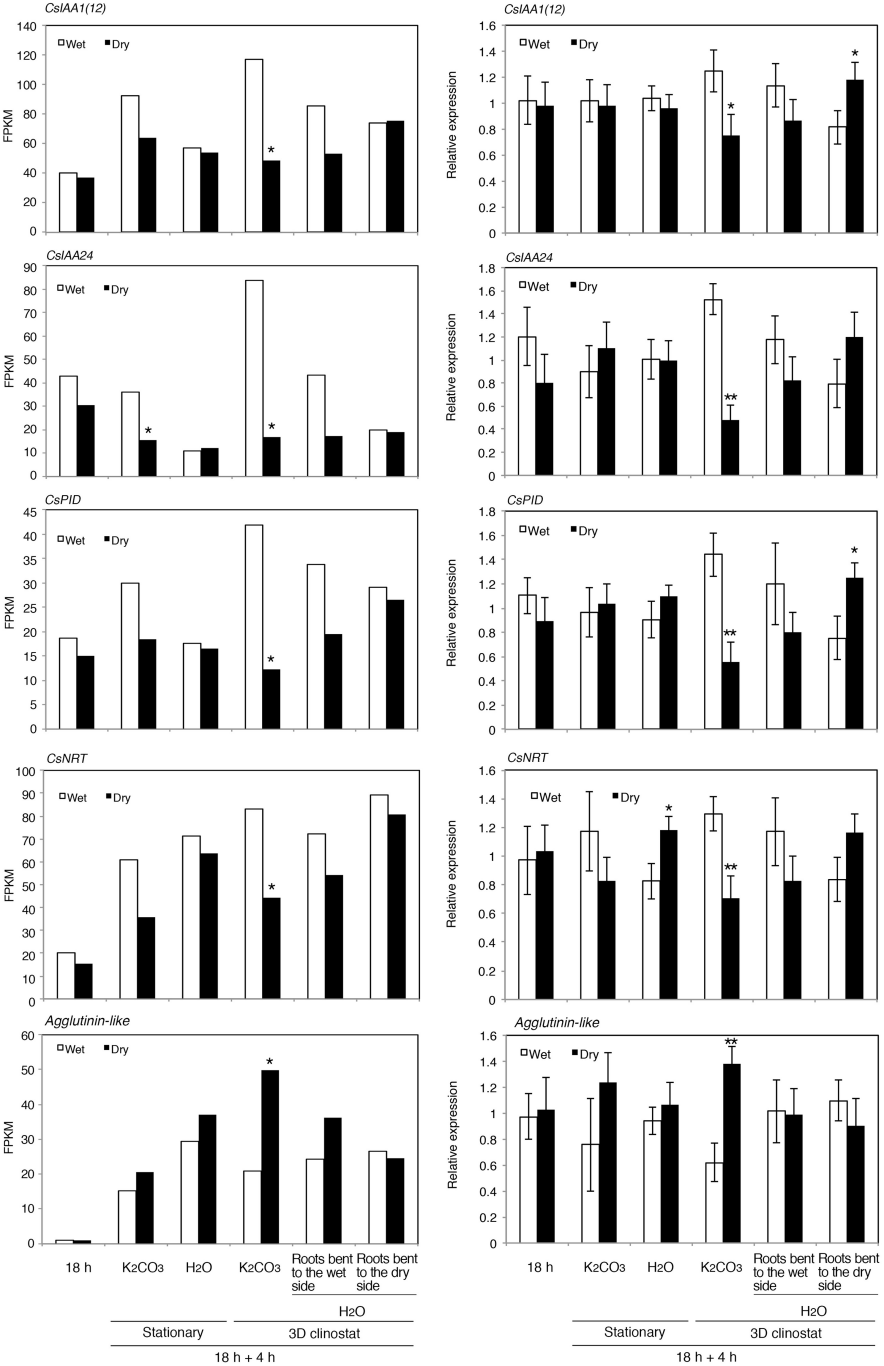
Expression of asymmetrically expressed genes in hydrotropically bending cucumber roots. FPKM (Fragments Per Kilobase of transcript per Million mapped reads) values representing mRNA levels from RNA-Seq (left) and relative expression levels from RT-qPCR (right) of *CsIAA1(12)*, *CsIAA24*, *CsPID*, *CsNRT*, and *Agglutinin-like* (Csa6M085120) are shown. Roots of 18-h-old cucumber seedlings grown in a vertical position (18 h), roots from 22-h-old cucumber seedlings exposed to a moisture gradient for 4 h induced by K_2_CO_3_ (K_2_CO_3_), and roots of 22-h-old cucumber seedlings incubated for 4 h under H_2_O-saturated conditions (H_2_O) were cut in half to produce the wet/water-absorbent plastic foam side and the dry/air side and used for RNA isolation. When the roots were exposed to a moisture gradient or incubated under H_2_O-saturated conditions, the seedlings were either under stationary conditions (stationary) or in a 3D clinostat (3D clinostat). After the roots incubated under H_2_O-saturated conditions were placed in a 3D clinostat, roots that bent to either the water-absorbent plastic foam side or the air side were separately harvested, and RNA samples were independently isolated. For the RNA-Seq results, a single asterisk indicates asymmetric gene expression (*Q*-value <0.05). For RT-qPCR, each data point represents the mean ± SD of five independent experiments. Statistically significant differences between the wet/water-absorbent plastic foam side and the dry/air side (determined by Student’s *t* test) are indicated by single (*P*<0.05) and double (*P*<0.01) asterisks.

Reverse transcription followed by quantitative polymerase chain reaction (RT-qPCR) analysis confirmed the expression patterns of several genes that were differentially expressed during root hydrotropism (Fig 3). Specifically, the expression patterns of *CsIAA1(12)*, *CsIAA24*, *CsPID*, *CsNRT* during the hydrotropic response in clinorotated cucumber roots exposed to a moisture gradient in the K_2_CO_3_ chamber determined by RT-qPCR were consistent with the results of RNA-Seq (Fig 3). In addition, the average relative expression levels of *CsIAA1(12)*, *CsIAA24*, *CsPID*, and *CsNRT* were greater in the concave side than in the convex side of bending roots in the H_2_O-saturated chamber under clinorotated conditions. However, the differences were much smaller or not detected compared with those of roots that showed obvious hydrotropic bending in the K_2_CO_3_ chamber.

RNA-Seq analysis suggested that three (Csa6G514890, Csa1G042560, and Csa2G006880) of the four genes that were preferentially expressed in the dry sides of roots that were clinorotated in the presence of moisture gradients were also included among the 16 genes preferentially expressed in the dry sides of roots exposed to a moisture gradient under stationary conditions (Table 1). Therefore, perhaps the expression of these three genes is induced by dry conditions and they are not necessarily involved in the induction of root hydrotropism. By contrast, the *Agglutinin-like* (Csa6G085120) gene was found among the four genes that were preferentially expressed in the dry sides of roots that were clinorotated in the presence of moisture gradients but not among the 16 genes that were preferentially expressed in the dry sides of roots under stationary conditions (Table 1). Therefore, we further examined the expression of *Agglutinin-like* gene by RT-qPCR and confirmed its preferential expression in the dry side of roots only when hydrotropism was induced (Fig 3). In addition, in contrast to the genes that were preferentially expressed in the wet side, the *Agglutinin-like* gene, which was preferentially expressed in the dry side, was not asymmetrically expressed in roots after a 4 h incubation under saturated H_2_O conditions.

**Fig 4:**
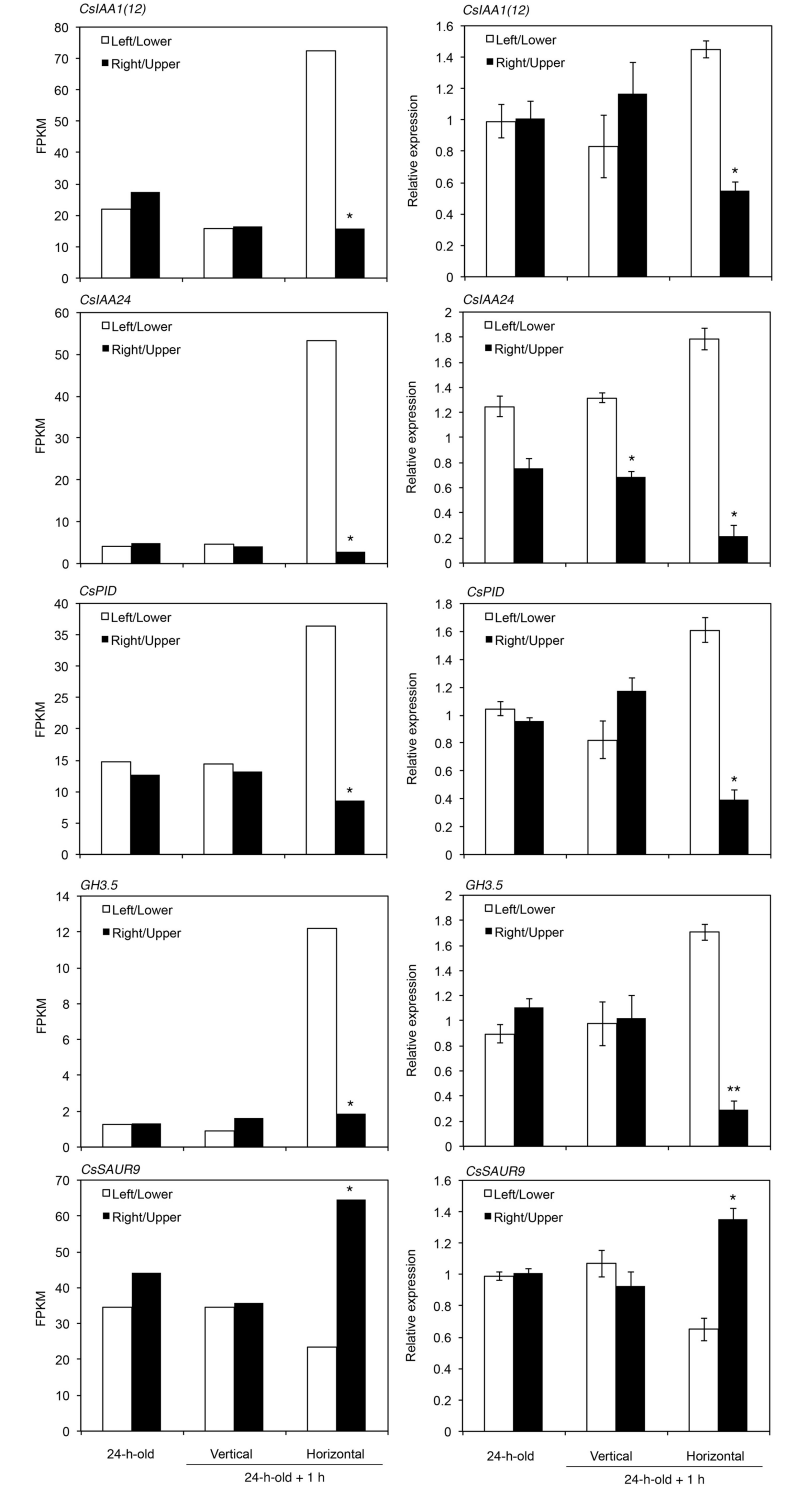
Expression of asymmetrically expressed genes in gravitropically bending cucumber roots. FPKM (Fragments Per Kilobase of transcript per Million mapped reads) values representing mRNA levels from RNA-Seq (left) and relative expression levels from RT-qPCR (right) of *CsIAA1(12)*, *CsIAA24*, *CsPID*, *CsGH3*.*5*, and *CsSAUR9* are shown. The roots of 24-h-old cucumber seedlings grown in a vertical position (24-h-old), seedlings placed in a vertical position for 1 h, and seedlings placed in a horizontal position for 1 h were cut in half, and RNA was isolated from the left/lower side and right/upper side. For the RNA-Seq results, a single asterisk indicates asymmetric gene expression (*Q*-value <0.05). For RT-qPCR, each data point represents the mean ± SD of three independent experiments. Statistically significant differences between the left/lower side and the right/upper side (determined by Student’s *t* test) are indicated by single (*P*<0.05) and double (*P*<0.01) asterisks.

We confirmed the differential expression patterns of *CsIAA1(12)*, *CsIAA24*, *CsPID*, *CsGH3*.*5*, and *CsSAUR9* during root gravitropism via RT-qPCR, and the results were consistent with the results of RNA-Seq, except for *CsIAA24* (Fig 4). RT-qPCR analysis showed that *CsIAA24* expression significantly differed (1.9-fold, *P*<0.05) between each side of the root in plants continuously grown in a vertical position, although the difference in *CsIAA24* expression between the lower and upper side of the root was much greater (8.2-fold, *P*<0.05) in the roots of seedlings grown in a horizontal position.

Cucumber *Like-AUX1 3* (*CsLAX3*), encoding auxin influx carrier protein, was included among the 16 genes found to be preferentially expressed in the dry side of the root grown under stationary conditions via RNA-Seq. Since root gravitropism requires the influx of auxin [27], we attempted to confirm the asymmetric expression of *CsLAX3* by RT-qPCR but failed to do so (S4 Fig). In cucumber roots, immunohistochemical analysis has shown that CsPIN5 localized to epidermal cells at the basal end of root accumulates more on the wet side than on the dry side [12]. However, the results of RNA-Seq and RT-qPCR did not show asymmetric accumulation of *CsPIN5* mRNA in roots (S4 Fig), suggesting that asymmetric accumulation of CsPIN5 proteins is post-translationally regulated. In Arabidopsis, *MIZ1* gene is required for the expression of hydrotropism in Arabidopsis roots [28]. To access the expression of the *MIZ1*-ortholog gene during hydrotropism in cucumber roots, we conducted a BLAST search and found nine *MIZ1*-like genes in cucumber. Phylogenetical analysis of cucumber MIZ1-like proteins revealed that the nucleotide sequence of Csa3G822470 would encode a protein belonging to the clade containing Arabidopsis MIZ1 (S5A Fig), suggesting that Csa3G822470 encodes a putative MIZ1-ortholog. It should be noted that the amino acid sequence (Csa3G822470) encoded by the opposite strand of the one encoding the putative MIZ1-ortholog protein was found in a BLAST Database for Chinese Long protein. Asymmetric expression of Csa3G822470 (*CsMIZ1*) could not be detected by RNA-Seq analysis (*Q*-values >0.99, S5B Fig). In addition, the *miz2* mutation in the *GNOM* gene causes a defect in root hydrotropism in Arabidopsis [29]. GNOM is an ARF guanine nucleotide exchange factor (ARF-GEF) proteins [30]. Phylogenetic analyis of ARF-GEF proteins suggested that Csa5G183270 in cucumber is a GNOM-ortholog in cucumber (S6A Fig). Asymmetric expression of Csa5G183270 (*CsGNOM*) could not be detected by RNA-Seq analysis (*Q*-values >0.99, S6B Fig).

**Fig 5:**
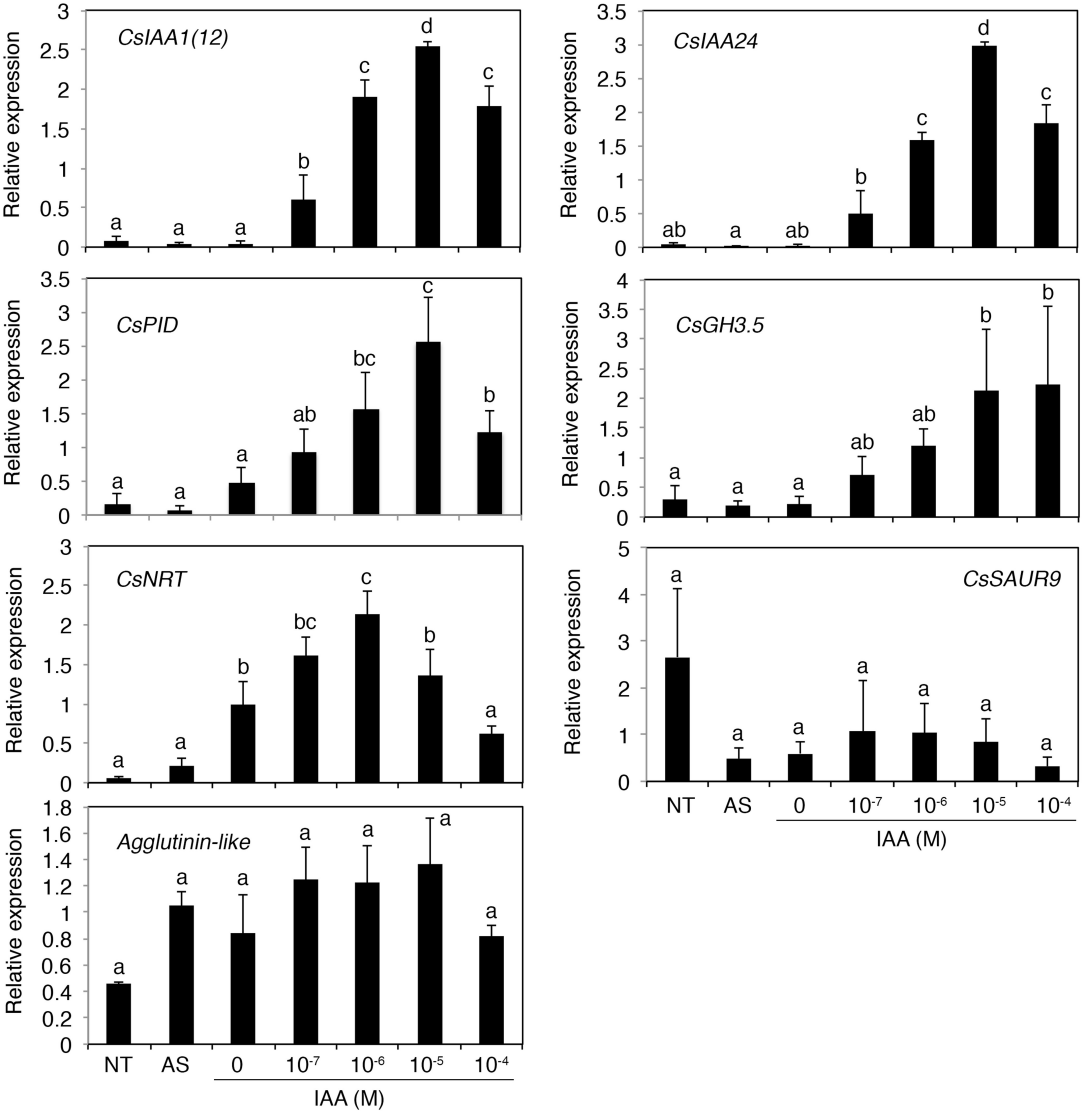
RT-qPCR to examine the auxin-inducible expression of asymmetrically expressed genes during hydrotropism and/or gravitropism in cucumber roots. Root segments excised from cucumber seedlings (No treatment), root segments from which auxin was depleted for 2 h (After auxin starvation), and root segments that were continuously treated for 2 h with the indicated concentration of auxin were subjected to gene expression analysis. Each data point represents the mean ± SD of three independent experiments. Different letters indicate statistically significant differences in relative expression levels (*P*<0.05), which were analyzed by one-way ANOVA and Tukey HSD tests.

Several genes that are asymmetrically expressed in roots during hydrotropism and/or gravitropism are auxin-inducible genes, such as *Aux/IAA*, *GH3*, and *SAUR*. Therefore, we examined the effect of auxin on the expression of these genes via RT-qPCR (Fig 5). Auxin treatment induced the expression of *Aux/IAA* and *GH3*, which are preferentially expressed in the concave side during both hydrotropism and gravitropism. Auxin treatment also increased the expression of *CsPID* and *CsNRT*, whose orthologs are induced by auxin treatments in Arabidopsis [31, 32]. On the other hand, auxin treatment did not increase the expression of *Agglutinin-like* and *CsSAUR9*, which are more highly expressed in the convex side than the concave side during hydrotropism and gravitropism, respectively (Figs 3 and 4).

### Expression analysis of auxin-inducible genes during hydrotropism of the de-tipped cucumber roots

**Fig 6:**
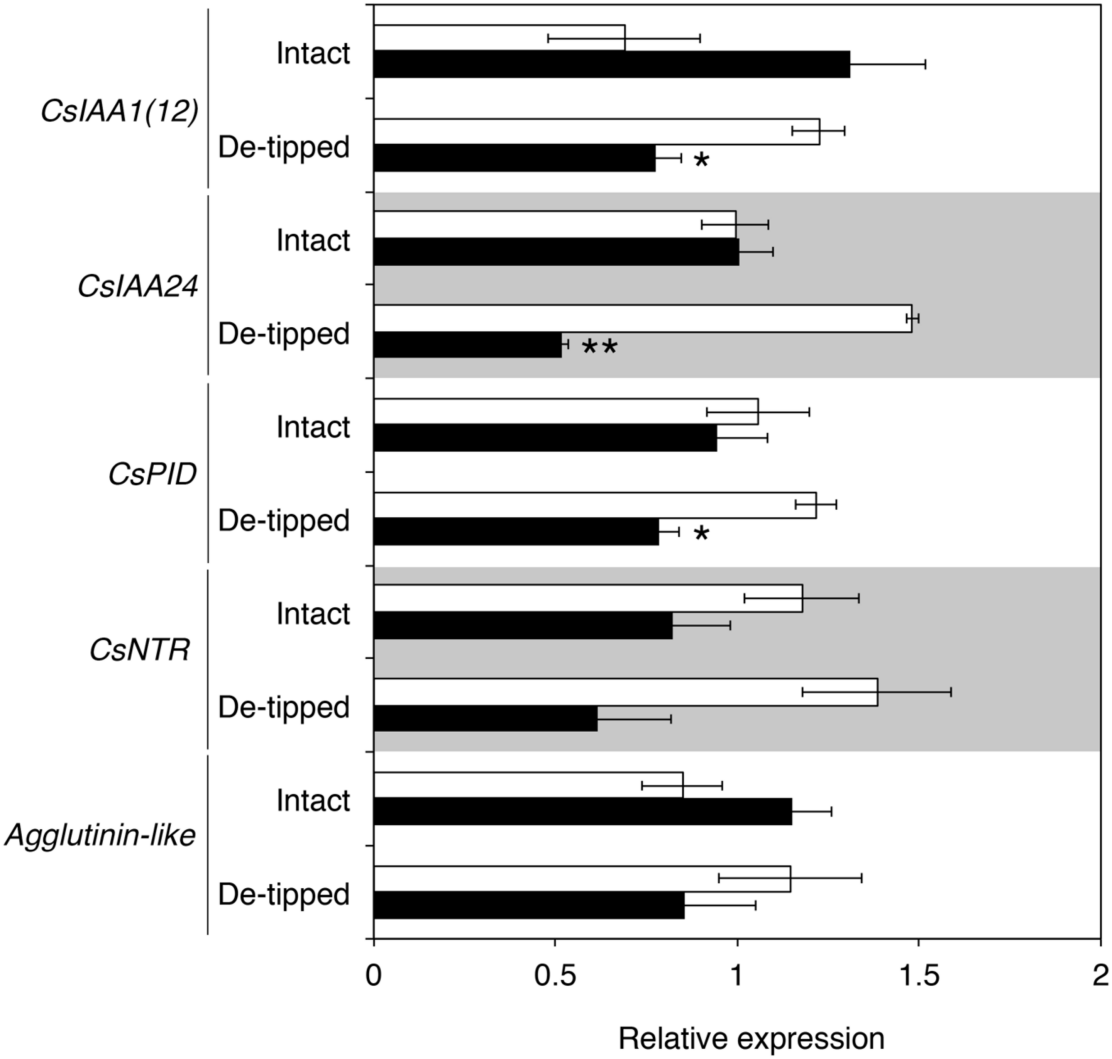
Effects of root tip removal on the expression of asymmetrically expressed genes during hydrotropism in intact cucumber roots. Roots in which the root tip was either not removed (Intact) or removed (De-tipped) were exposed to a moisture gradient induced by K_2_CO_3_ under stationary conditions for 4 h and cut in half, RNA was isolated from the wet side and the dry side. Each data point represents the mean ± SD of three independent RT-qPCR experiments. Statistically significant differences in relative expression levels between the wet and dry sides (determined by Student’s *t* test) are indicated by single (*P*<0.05) and double (*P*<0.01) asterisks.

In addition to *CsIAA1(12)*, RNA-Seq identified several other auxin-inducible genes that are asymmetrically expressed during both hydrotropism and gravitropism in cucumber roots. We examined the expression of these auxin-inducible genes in cucumber roots in which hydrotropism was induced by removing the root tip under stationary conditions. When intact cucumber roots were exposed to a moisture gradient under stationary conditions, *CsIAA1(12)*, *CsIAA24*, and *CsPID* mRNA levels in the wet side were not significantly different from those in the dry side (Fig 6). On the other hand, in de-tipped roots exposed to a moisture gradient for 4 h, *CsIAA1(12)*, *CsIAA24*, and *CsPID* mRNA levels were greater in the wet side than the dry side (Fig 6). These results suggest that root hydrotropism is induced by the removal of the root tip in cucumber seedlings under stationary conditions due to asymmetric auxin redistribution, which occurs in response to a moisture gradient. However, we failed to detect asymmetric expression of *CsNRT* and *Agglutinin-like* in de-tipped roots exposed to a moisture gradient under stationary conditions. These results suggest that the gene expression patterns in hydrotropically bending de-tipped roots under stationary conditions are not completely identical to those in hydrotropically bending clinorotated roots of intact seedlings.

## Discussion

### Gravitropism suppresses hydrotropism in cucumber roots

Root hydrotropism was first reported over one hundred years ago [33–35]. Analysis using the pea *ageotropum* mutant, which is defective in root gravitropism, has clearly shown that roots bend toward the wetter side [2]. When wild-type pea and cucumber roots were exposed to a moisture gradient, clinorotation induced hydrotropism, although they did not exhibit hydrotropism under stationary conditions [3–5]. These results indicate that gravitropism suppresses hydrotropism in pea and cucumber roots. The root cap columella cells contain sedimenting amyloplasts (or statoliths), which are required for full gravitropic sensitivity [36]. Therefore, root cap cells are thought to sense gravity. In contrast to root gravitropism, removing root columella cells via laser ablation failed to inhibit hydrotropism in Arabidopsis roots [17]. Moreover, surgically removing the root tip (0.2 mm in length) also failed to inhibit hydrotropism in rice roots [7]. These results indicate that root tissues other than the root cap can sense a moisture gradient. In contrast to Arabidopsis and rice roots, which exhibit hydrotropism under stationary conditions, cucumber roots hardly do so [5]. In the current study, we showed that removing the root tip induced hydrotropism in cucumber roots under stationary conditions (Fig 1). This finding suggests that the root tip inhibits hydrotropism via gravity responses in cucumber roots and that root tissue(s) other than the root tip can sense a moisture gradient.

Root columella cells respond to gravistimulation by re-localizing AtPIN3/AtPIN7 auxin efflux carriers to the lower sides of cells, followed by auxin transport to the lower sides of roots [9, 10]. Therefore, removing the root tip would nullify the plant’s ability to regulate auxin transport in response to gravistimulation. As a result, parts of the root other than the root tip of cucumber, as well as Arabidopsis and rice, can respond to a moisture gradient by bending to the wet side. In this study, we showed that cucumber roots without the tip can exhibit hydrotropism, which depends on auxin transport (Fig 2). In addition, when cucumber roots without the tip were exposed to a moisture gradient, several auxin-inducible genes were predominantly expressed in the wet side, although some genes that were asymmetrically expressed during hydrotropism in intact roots were not asymmetrically expressed in these tip-less roots (Fig 6). These results suggest that root tissues other than the root tip are responsible for sensing a moisture gradient and regulating auxin transport to induce asymmetric auxin distribution.

In Arabidopsis, AtPIN2 auxin efflux carriers predominantly accumulate in the lower sides of gravitropically bending roots [37]. This asymmetric accumulation of AtPIN2 is thought to be regulated by the levels of auxin derived from root columella cells in response to gravistimulation [37, 38]. Recently, CsPIN5 protein, a AtPIN2 ortholog in cucumber, was shown to accumulate predominantly in the wet side of the root during hydrotropism, as well as in the lower side during gravitropism [12]. Therefore, it is likely that signals transmitted from the root tip in response to gravity interfere with the hydrotropic responses in the elongation region of cucumber roots. CsPIN5-mediated auxin transport due to gravitropic and hydrotropic responses could compete for the interference between the two tropisms. Pronounced hydrotropism was observed in de-tipped roots (Fig 1), suggesting that the hydrotropic response in the transition and elongation zones might directly regulate the accumulation or asymmetric expression of CsPIN5 in response to moisture gradients.

There are at least 2 possibilities to explain how de-tipped roots obtain auxin for hydrotropism. One possibility is that the root division zone already has enough auxin to express hydrotropism and the asymmetrical distribution of CsPIN5 efflux carriers in response to moisture gradients induces asymmetric auxin redistribution. The other possibility is that an unidentified lateral auxin transport pathway not involving the root tip functions to supply auxin to the bending region of roots. It has been suggested that wounding responses induce relocalization of AtPIN1 in Arabidopsis stems as well as putative PsPIN1 in pea epicotyls to the lateral side of vascular cells to transport auxin for the regeneration of vascular tissues [39, 40]. Therefore, the removal of root-tip might induce relocalisation of CsPIN efflux carriers to the pith of roots to deliver auxin to the cortex and epidermis of cucumber roots.

In Arabidopsis, root cortical cells are important for hydrotropic responses [17] whereas lateral root cap and root epidermal cells are important for gravitropic responses [41]. Hydrotropism in Arabidopsis roots was not inhibited by auxin transport inhibitors but gravitropism was inhibited [13]. In cucumber roots, the epidermis may be important for hydrotropic responses as well as gravitropic responses because auxin transport inhibitors inhibited both hydrotropism and gravitropism and because CsPIN5 signals in epidermal cells were stronger than in the cortex [12].

### Asymmetrically expressed genes during hydrotropism and gravitropism in cucumber roots

Transcriptome analysis was previously used to identify genes that are asymmetrically expressed during hypocotyl phototropism and hypocotyl gravitropism in *Brassica oleracea* seedlings using Affymetrix Arabidopsis whole-genome arrays [42]. Even though hybridization signals from only 4,500 out of 24,000 genes were observed because cRNAs derived from *B*. *oleracea* were heterogeneously hybridized to the Arabidopsis arrays, the authors were able to identify auxin-inducible genes (such as *SAUR50* and *GH3*.*5*) that are predominantly expressed in the convex side of the hypocotyl [42]. Microarray analysis in rice showed that auxin-inducible *SAUR*s and *Aux/IAA*s are preferentially expressed in the convex side [43]. Moreover, auxin-inducible genes including *SAUR*s and *Aux/IAA*s, are predominantly expressed in the convex side of gravistimulated Arabidopsis stems, as revealed using an Arabidopsis microarray [44]. These transcriptome analyses of gravistimulated aerial organs indicated that auxin-inducible genes including *SAURs* are predominantly expressed in the convex side (lower side) of the organ.

In the current study, we comprehensively identified genes that are asymmetrically expressed during hydrotropism and gravitropism in cucumber roots. GO analysis of the identified genes revealed that, during both root tropisms, auxin-inducible genes including *Aux/IAA* genes are predominantly expressed in the concave side of the bending root (Tables 1 and 2). Of these, auxin-inducible genes (*CsIAA1(12)*, *CsIAA24*, and *CsPID*) that were asymmetrically expressed during root gravitropism as well as during root hydrotropism were asymmetrically expressed during the hydrotropic response in de-tipped cucumber roots under stationary conditions (Fig 6). Therefore, the asymmetric expression of *CsIAA1(12)*, *CsIAA24*, and *CsPID* is characteristic of bending responses during root tropism. These results also suggest that auxin plays an important role in regulating the direction of root bending in cucumber seedlings during both root tropisms. These conclusions are supported by the finding that auxin transport inhibitors suppress hydrotropism and gravitropism in cucumber roots [12]. In contrast to the genes more highly expressed in the concave side, genes that were more highly expressed in the upper side during gravitropism differed from those in the dry side during hydrotropism in cucumber roots (Tables 1 and 2). Therefore, it is likely that physiological property of the convex side of gravistimulated roots differs from that of hydrostimulated roots in cucumber seedlings.

The results indicate that *SAUR9* mRNA is much more highly expressed in the upper side (convex side) of the cucumber root than in the lower side (concave side) (Fig 4). *SAUR* mRNAs are more highly expressed in the lower side (convex side) of aerial organs of Arabidopsis and rice than in the upper side (concave side) [42–44]. SAUR proteins promote cell elongation by directly inhibiting PP2C-D phosphatase, which activates plasma membrane H^+^-ATPase [45]. Therefore, the greater expression of *SAUR* genes in the convex versus concave side during gravitropism in cucumber roots, as well as Arabidopsis hypocotyls, Arabidopsis stems, and rice shoots, is consistent with the greater cell elongation observed in the convex versus concave side. In addition, our results suggest that *SAUR9* expression is not auxin-inducible in cucumber roots (Fig 5). This property might allow *SAUR9* to be more highly expressed in the convex side than the concave side of cucumber roots during gravitropism, as auxin-inducible genes such as *Aux/IAA*s were predominantly expressed in the concave side. These results suggest that *SAUR9*, whose expression is not induced by auxin, might play a role in promoting cell elongation in the convex side of the cucumber root during gravitropism. Differences in elongation in response to auxin between root cells and shoot cells are thought to underlie differences in the direction of curvature between roots and shoots [8, 46]. In addition to these differences, it is likely that the predominant expression of *SAUR9* in the upper side of the cucumber root contributes to the differences in the direction of curvature between roots and shoots.

## Conclusion

In this study, we showed that auxin transport and redistribution are required for the induction of hydrotropism in cucumber roots, but these processes are independent of root-tip-mediated auxin transport. The root tip, in which graviresponsive auxin transport occurs, is inhibitory to the hydrotropic response. In addition, RNA-Seq experiments identified genes that are asymmetrically expressed during hydrotropism and gravitropism in cucumber roots. GO analysis revealed that genes that are much more highly expressed in the concave side than the convex side during both tropisms are specifically enriched in the category auxin-inducible genes. Our results suggest that removing the root tip eliminates graviresponsive auxin transport and redistribution, but root-tip-independent auxin transport is still induced in response to moisture gradients, which results in the asymmetric expression of several auxin-inducible genes. These results reveal that cucumber roots express a distinct hydrotropic response that differs from the auxin transport independent hydrotropism in Arabidopsis roots.

## Supporting information

S1 Fig Chambers used to form a moisture gradient to induce hydrotropism in cucumber roots. Schematic drawings of water-absorbent plastic foam (A) and the chamber (B), and photograph of the chamber (C) are shown. Seven cucumber seeds were inserted into a crack created in a block of water-absorbent plastic foam (upper depth 10 mm × lower depth 17.5 mm × H10 mm × W49 mm) so that the 2 mm of the micropylar end side of each seed was extruded from the block. A metallic pipe with small holes connected to a port on the outside of the container (for the injection of water) was inserted into the plastic foam. The plastic foam was placed at one side of the plastic container so that plant roots could emerge into the center of the plastic container and grow into the air after germination. A plastic syringe fitted with a tap and a tube (to draw water from the water reservoir) was connected to the port in the plastic container. The plastic foam was supplied with 9 mL of distilled water by pushing a plunger to initiate imbibition of the cucumber seeds. Extra Thick (2.45 mm) Blot Absorbent Filter Paper (70 × 41 mm) (Bio-Rad) with a hole in the center was held at the opposite side of the plastic foam in the container between the bolt and the wall of the container. At 18 h after the start of imbibition, 6 mL of saturated K_2_CO_3_ solution was supplied to the filter paper using a plastic syringe to form a moisture gradient in the container.
(EPS)

S2 Fig Recovery of root gravitropism after root tip removal in cucumber seedlings. 24-h-old cucumber seedlings with root tip was removed (De-tipped in 0.5 mm or 1.0 mm) or not removed (Intact) were placed in horizontal positions. After 0, 1, 2 and 4 h, root curvature (A) and length (B) were measured. In panel B, incremental differences in root length from the average of root length at 0 h in each experiment were calculated and represented as growth. Experiments (using 7 seedlings) were repeated three times, and the mean ± SE was calculated. Different letters indicate statistically significant differences (*P*<0.01), which were analyzed by one-way ANOVA and Tukey HSD tests.
(EPS)

S3 Fig Time-course study of root gravitropism in cucumber seedlings. Roots that were aeroponically grown in a vertical position in humid air in a plastic container for 24 h were exposed to a gravistimulation by placing them in a horizontal position (plots in black). For a control, the roots of 24-h-old cucumber seedlings were continuously held in a vertical position (plots in white). Single asterisk and double asterisks indicate the root curvature values in a horizontal position that significantly differed from that in a vertical position at *P*<0.05 and *P*<0.01, respectively.
(EPS)

S4 Fig Expression of *CsLAX3* and *CsPIN5* during cucumber root hydrotropism. FPKM (Fragments Per Kilobase of transcript per Million mapped reads) values representing mRNA levels from RNA-Seq (left) and relative expression levels from RT-qPCR (right) are shown. The roots of 18-h-old cucumber seedlings grown in a vertical position (18 h), and 18-h-old cucumber seedlings exposed to a moisture gradient induced by K_2_CO_3_ (K_2_CO_3_) and H_2_O (H_2_O), under stationary conditions (stationary) and in a 3D clinostat (3D clinostat) for 4 h were cut in half, and the wet side and the dry side were subjected to RNA isolation. To amplify cDNA by PCR, the CsLAX3_F_+_853 (5’-TTTGGGGATGACCTCTTG-3’) and CsLAX3_R_+_1008 (5’-AGCTATTCGTCTCATGCACC-3’) primers for *CsLAX3* and the CsPIN5_F_+_830 (5’-ACACCAATAGTTTTCAAGGC-3’) and CsPIN5_R_+_988 (5’-GAAGAAGCTGAAAACATGGG-3’) primers for *CsPIN5* were used.
(EPS)

S5 Fig Expression of the *CsMIZ1* gene during cucumber root hydrotropism. Phylogenetic analysis of MIZ1-like proteins (A). Amino acid sequences of MIZ1 domain (DUF617 domain) were aligned using ClustalW (http://clustalw.ddbj.nig.ac.jp/index.php?lang=en) and phylogenetically analyzed by the neighbor-joining method. The number at each node indicates the bootstrap support from 1,000 replicates. Protein IDs that are begin with AT, Cs, GLYMA, Solyc, OS and Zm are respectively Arabidopsis, cucumber, soybean, rice and maize proteins. MIZ1-like proteins (PP1S3_412V6, PP1S20_106V6 and PP1S3_412V6) of moss (*Physcomitrella patens* subsp. patens) were used as an outgroup. FPKM (Fragments Per Kilobase of transcript per Million mapped reads) values represent the mRNA levels of Csa3G822470 (*CsMIZ1*) from RNA-Seq (B). The roots of 18-h-old cucumber seedlings grown in a vertical position (18 h), and 18-h-old cucumber seedlings exposed to a moisture gradient induced by K_2_CO_3_ (K_2_CO_3_) and H_2_O (H_2_O), under stationary conditions (stationary) and in a 3D clinostat (3D clinostat) for 4 h were cut in half, and the wet side and the dry side were subjected to RNA isolation. Asymmetric expression in roots was not detected (*Q*-values >0.05).
(EPS)

S6 Fig Expression of the *CsGNOM* gene during cucumber root hydrotropism. Phylogenetic analysis of ARF guanine nucleotide exchange factor (AFR-GEF) proteins (A). Amino acid sequences of Sec7 domain were aligned using ClustalW (http://clustalw.ddbj.nig.ac.jp/index.php?lang=en) and phylogenetically analyzed by the neighbor-joining method. The number at each node indicates the bootstrap support from 1,000 replicates. Protein IDs that are begin with AT, Cs, GLYMA, Solyc, OS and Zm are respectively Arabidopsis, cucumber, soybean, rice and maize proteins. SEC7 (NP_010454) of yeast (*Saccharomyces cerevisiae*) was used as an outgroup. FPKM (Fragments Per Kilobase of transcript per Million mapped reads) values represent the mRNA levels, of Csa5G183270 (*CsGNOM*) from RNA-Seq (B). The roots of 18-h-old cucumber seedlings grown in a vertical position (18 h), and 18-h-old cucumber seedlings exposed to a moisture gradient induced by K_2_CO_3_ (K_2_CO_3_) and H_2_O (H_2_O), under stationary conditions (stationary) and in a 3D clinostat (3D clinostat) for 4 h were cut in half, and the wet side and the dry side were subjected to RNA isolation. Asymmetric expression in roots was not detected (*Q*-values >0.05).
(EPS)

S1 Table Gene-specific primers used in qRT-PCR analysis. (XLSX)

S2 Table Mapping of RNA-Seq reads obtained from cucumber roots exposed to various water conditions onto the cucumber genome sequences. (XLSX)

S3 Table Significantly enriched GO terms among preferentially expressed genes in the wet side in the roots of cucumber seedlings exposed to a moisture gradient in a 3D clinostat for 4 h. (XLSX)

S4 Table Significantly enriched GO terms among preferentially expressed genes in the dry side of the roots in cucumber seedlings that were exposed to the moisture gradient under stationary conditions for 4 h. (XLSX)

S5 Table Mapping of RNA-Seq reads obtained form cucumber roots exposed to gravistimulation onto the cucumber genome sequences (cucumber_ChineseLong_v2_genome.fa). (XLSX)

S6 Table Significantly enriched GO terms among preferentially expressed genes in the lower side of roots in cucumber seedlings that were exposed to gravistimulation for 1 h. (XLSX)
